# The Effect of Age on the Regenerative Potential of Human Eyelid Adipose-Derived Stem Cells

**DOI:** 10.1155/2018/5654917

**Published:** 2018-03-19

**Authors:** Mingqi Zhang, Zhuoshi Wang, Yan Zhao, Lirong Zhang, Ling Xu, Liu Cao, Wei He

**Affiliations:** ^1^Key Laboratory of Medical Cell Biology, Institute of Translational Medicine, China Medical University, Shenyang, Liaoning, China; ^2^Clinical Research Center, He Eye Hospital of He University, Shenyang, Liaoning, China; ^3^Key laboratory of Stem Cell Research, College of Basic Medicine, He University, Shenyang, Liaoning, China

## Abstract

Human eyelid adipose-derived stem cells (HEASCs) are a new source of autologous mesenchymal stem cells, which are derived from neuroectoderm and potentially applied in the tissue regeneration and cell therapies. Based on the prevalence of blepharoplasty in Asia and the availability of HEASCs, we investigated the effect of donor age on their characteristics and regenerative potential of HEASCs in vitro. The HEASCs were isolated from patients of three groups: (1) <20 years (*n* = 4), (2) >20 years, <45 years (*n* = 5), and (3) >55 years (*n* = 4). For each group, the proliferative capacity, colony-forming ability, surface markers, differentiation ability, wound healing function, and secreted protein were contrastively evaluated and quantified for statistical analysis. It was found that HEASCs were successfully isolated and cultured by an explant culture method. The proliferative rates, osteogenic and chondrogenic differentiation potentials, wound healing ability, and the expression of TGF-*β*1 and fibronectin protein of HEASCs significantly decreased as age increased. However, the expression of CD90 antigen and the adipogenic differentiation showed an age-related increase in HEASCs. As many degenerative diseases increase in prevalence with age, the age-related changes of the HEASCs proliferation potential, differentiation capacity, and wound healing ability should be taken into account whenever they are intended for use in research or cytotherapy.

## 1. Introduction

Mesenchymal stem cells (MSCs) are one of the most prominent stem cell types in the translational medicine due to their regenerative potential and paracrine effect for treating different kinds of diseases [1–3]. The use of MSCs in clinical trials is more than 600 trials listed in the clinical trials database (http://www.clinicaltrials.gov/, accessed on July 2017). Although cell therapy with bone marrow-derived stem cells (BMSCs) is under investigation in preclinical studies and clinical trials, the use of adipose-derived stem cells (ASCs) involves less religious and ethical concerns than using bone marrow-derived stem cells because they are easily obtainable from medical waste [4, 5]. Therefore, there has been the growing interest in applying ASCs as a potential cell source in stem cell-based therapy and tissue engineering.

Previous studies have reported the successful isolation and culture of multipotent stem cells from eyelid adipose tissue or orbital adipose tissues [6–8]. Human eyelid adipose-derived stem cells (HEASCs) are novel MSCs and originated from the neural crest source. In addition, HEASCs are also similar to BMSCs in the growth potential and surface marker expression [7, 9]. In addition, HEASCs lacked immunogenicity and were safe and well-tolerated after intravenous injections and topical administration in previous studies [10, 11]. Thus, it is possible that HEASCs may be an excellent candidate therapeutic adipose-derived stem cell type for treating complicated diseases.

In a wide range of animal model studies, it has been demonstrated that HEASCs can be successfully applied in repairing neurological disorders [12, 13]. In general, cell-based therapy involves the delivery of expanded cell populations to the degenerative tissues [14] or the mobilization of endogenous progenitor cells capable of proliferating and differentiating into required tissues through the paracrine function [15]. The regenerative potential of host tissues may be restricted by an age-related decline in progenitor populations [16, 17]. In this case, an external source of neural crest stem cells may meet the needs of regenerative damaged tissue cells. Although HEASCs can be easily obtained during blepharoplasty for an entropic and baggy lid and can be a good source of isolating stem cells for tissue repair purposes, more information concerning the effect of donor age on cell properties is essential for clinical applications and cell-based therapy. However, the research of the effect of donor age on HEASCs was limited [18]. Therefore, the aim of the study is to investigate how the morphology, cell surface markers, proliferative potential, differentiation capacity, wound healing ability, and secreted wound healing-related factors of HEASCs were affected by donor age.

## 2. Materials and Methods

### 2.1. Study Design

This study was conducted from June in 2015 to December in 2016. Human eyelid adipose tissues were obtained from 13 systematically healthy donors who underwent blepharoplastic surgery. The eyelid adipose tissues were divided into three groups based on the donor's age: Group A (age range, 7–18 and mean age, 14.25 ± 4.99, *n* = 4), Group B (age range, 21–45 and mean age, 27 ± 10.04, *n* = 5), and Group C (age range, 55–75 and mean age, 63 ± 8.64, *n* = 4). The protocol for this study was approved by the Research Committee of He Eye Hospital of He University and conformed to the principles outlined in the Declaration of Helsinki. All participants have provided their written informed consent to participate in this study.

### 2.2. Isolation and Culture of HEASCs

All eyelid adipose tissue samples were processed under the same conditions. After surgical harvesting, redundant adipose tissues were placed in Hanks' Balanced Salt Solution (HBSS, Gibco) to rinse three times and minced into pieces about 1 × 1 mm^2^ after removing of the fibrous tissues and visible blood vessels. The piece of tissue was adopted to explant culture and attached to 6 cm culture dish and inverted for 1 h. After that, the tissues were maintained in the human adipose-derived mesenchymal stem cell growth medium (Cyagen Biosciences, China) at 37°C with 5% CO_2_, and the medium was changed every 2 days. After cell growth, the tissues were moved into a new culture dish. Once adherent cells reach 90%–100% confluence, they were detached with 1 : 1 TrypLE Express (Invitrogen, China) and replated at 1 : 3 under the same culture conditions.

Primary culture of HEASCs was designated as “passage 0.” To prepare cells for experiments, they were passaged five times.

### 2.3. Cell Proliferation Assays

HEASCs at passage 5 were plated in 96-well plates at a density of 3000 per well, and cell proliferative rate was assayed using the Cell Counting Kit-8 (CCK-8) according to the manufacturer's protocol. In brief, 10 *μ*l of CCK-8 solution was added to each well and the samples were incubated for 3 h. The absorbance was measured at 490 nm. This experiment was repeated three times.

For the colony-forming unit (CFU) assay, HEASCs were plated at a density of 100 cells in 60 mm plates and allowed to grow for 14 days. Cultures were terminated and stained with crystal violet for colony visualization. For colony number counting, only aggregates of >50 cells were scored as colonies; colonies < 50 cells in number and/or faintly stained were excluded. The colonies were counted manually under an inverted microscope.

### 2.4. Flow Cytometry Analysis

After HEASCs were harvested, nonspecific bindings were blocked and the cells were stained with different fluorochrome-conjugated monoclonal antibodies specific for CD105, CD44, CD73, CD90, CD31, CD34, and CD45 (all antibodies purchased from BD Biosciences). Isotype-matched antibodies were used as controls. Samples were analyzed on a FACSCalibur, and data acquisition and analysis were performed using CellQuest (BD Biosciences).

### 2.5. Multilineage Differentiation

#### 2.5.1. Adipogenic Differentiation

Adipogenic differentiation was induced by culturing HEASCs for 2 weeks in the adipogenic differentiation medium (Gibco) and assessed by Oil Red O (Sigma-Aldrich) staining as an indicator of intracellular lipid accumulation. Prior to staining, the cells were fixed with 70% ethanol for 15 min at room temperature and then stained with fresh Oil Red O for 15 min at room temperature. Excess stain was removed by washing with 70% ethanol and then distilled water to visualize lipid droplets. To elute the Oil Red O solution, 100% isopropanol was added for 1 h on an orbital shaker and the optical density of the solution was measured at 490 nm.

#### 2.5.2. Osteogenic Differentiation

Osteogenic differentiation was induced by culturing HEASCs for 3 weeks in the osteogenic differentiation medium (Gibco) followed by examining extracellular matrix calcification by Alizarin Red S (Sigma-Aldrich) staining. For Alizarin Red S staining, cells were fixed with 70% ethanol and washed with distilled water, then incubated in 2% Alizarin Red S solution for 15 min at room temperature. The cells were washed five times in PBS and then fixed with ice-cold 70% ethanol for 1 h. The Alizarin Red S solution was extracted by incubation of the cells in cetylpyridinium chloride for 1 h. The optical density of the solution was read at 550 nm.

#### 2.5.3. Chondrogenic Differentiation

HEASCs were trypsinized, counted, and centrifuged into cell pellets (1.2 × 10^5^ per pellet) in 15 ml conical tubes (BD Falcon). Chondrogenic differentiation medium (Gibco) was gently overlaid so as to not detach the cell nodules, and the culture was maintained in the chondrogenic differentiation medium for 3 weeks. Afterwards, the pellets were fixed and frozen in O.C.T. (Tissue-Tek, Sakura, Zoeterwoude, Netherlands). The pellets were sectioned, fixed with 4% polymethanol, stained against Alcian blue stain, and further stained with hematoxylin-eosin stain (Sigma-Aldrich).

### 2.6. Real-Time Polymerase Chain Reaction

Total cellular RNA was isolated from the differentiated cells using a TRIzol reagent (Invitrogen) at day 21 to determine the expression of multilineage-associated genes. For each run, water was used as a negative control. The reaction product was quantified with a relative quantificative tool. The reactions were performed under the following cycling conditions: 95°C for 10 min, 45 cycles of 95°C for 15 s, and 60°C for 1 min. All experiments were conducted three times. For each sample, copy numbers of target gene were divided by those of GAPDH to normalize for target gene expression, thus avoiding sample-to-sample differences in RNA quantity. Primers used for real-time RT-polymerase chain reaction are listed in Table 1.

### 2.7. Condition Medium (CM) of HEASCs Collection

The HEASCs were plated in 75 cm^2^ flasks. After getting confluence, the medium was changed to 10 ml per flask of DMEM/F12 and incubated for 48 hours. Then, supernatants were collected to filter and centrifuge at 2000*g* for 30 min. The supernatant was developed as HEASCs-CM.

### 2.8. Human Corneal Epithelial Cell Wound Healing Assay

To evaluate the function of HEASCs-CM, human corneal epithelial cell line (Guangzhou, China) was seeded in 12-well plates at 1 × 10^5^ cells per well and incubated at 37°C with 5% CO_2_ until forming a confluent monolayer in the DMEM/F12 medium containing 10% fetal bovine serum (FBS) (Gibco). The confluent layer was scratched with a 100 *μ*l sterile pipette tip after washing with DPBS for 3 times. Then the culture medium was replaced with HEASCs-CM. Image (*n* = 3 per treatment) from the center of the wells was taken with an IX51 Olympus microscope with a DPI 7.2 digital camera and time 0, 24 h, and 48 h after scratching. The areas of wound healing were analyzed and compared with the ImageJ software.

### 2.9. Measurement of Secreted Wound Healing-Related Factors in HEASCs-CM

The HEASCs-CM from all donor age group was collected to evaluate the secreted protein using human enzyme-linked immunosorbent assay (ELISA). ELISA was performed using a human platelet-derived growth factor-BB (PDGF-BB), human transforming growth factor *β*1 (TGF-*β*1), human vascular endothelial cell growth factor (VEGF), human cellular fibronectin (cFn), human collagen type I (Col I), human interleukin 8 (IL-8) ELISA kit (CUSABIO, China), and their protocols. As a reference for quantification, a standard curve was established by a serial dilution for PDGF-BB protein (0.156 ng/ml–10 ng/ml), TGF-*β*1 protein (0.78 ng/ml–50 ng/ml), VEGF protein (31.25 pg/ml–2000 pg/ml), cFn protein (12.5 ng/ml–800 ng/ml), Col-1 protein (1.56 ng/ml–100 ng/ml), and IL-8 protein (31.25 pg/ml–2000 pg/ml).

### 2.10. Statistical Analysis

All data collected were presented as a mean ± standard deviation (SD). One-way analysis of variance with Bonferroni post hoc multiple comparison test was used to determine possible significant differences (*P* < 0.05) between groups. Data analysis was performed using GraphPad Prism 5.0. All experiments were performed in triplicate and repeated on at least three separate occasions.

## 3. Results

### 3.1. Proliferative Activities of HEASCs

**Figure 1:**
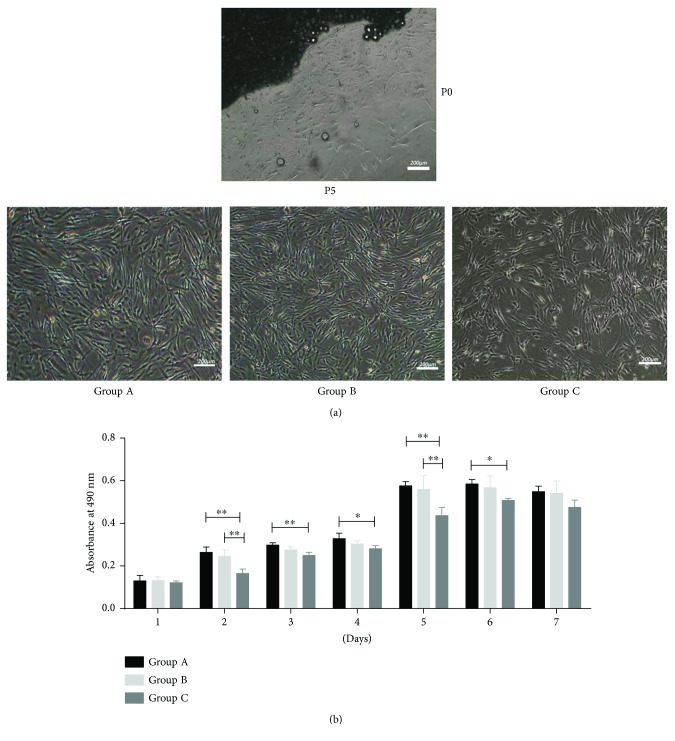
Proliferative activities of HEASCs in different groups. (a) Morphology of HEASCs. Scale bar = 200 *μ*m. (b) HEASCs from all groups showed increased proliferation over the time course. The effect of aging on the cellular proliferation of HEASCs showed the most significant decline at 5 days. The values are the mean ± SD. ^∗^*P* value < 0.05. ^∗∗^*P* value < 0.01.

**Figure 2:**
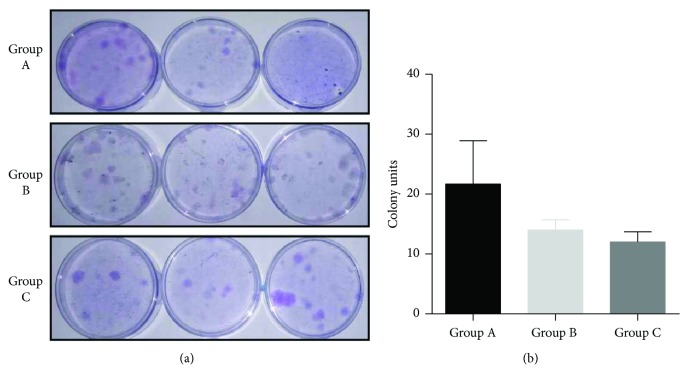
Comparison of colony-forming unit (CFU) efficiency of HEASCs. (a) Representative figures showed the colony numbers of HEASCs from three groups (14 d, ×20). (b) The graph represented a statistical difference in the total colony number in donor age groups. Results expressed as mean ± SD.

The HEASCs successfully outgrew from all donor age eyelid adipose tissues after 6–20 days of explant culture. In culture, the cell populations isolated from all eyelid tissues were capable of forming adherent cells, a characteristic of other stromal stem cell populations, and retaining their bipolar shape (Figure 1(a)). The proliferative capacities of all cells were assessed at passage five at the following time points: 1, 2, 3, 4, 5, 6, and 7 days. The HEASCs from Group C demonstrated a significantly lower proliferative rate at days 2, 3, 4, 5, and 6 (*P* < 0.05, or *P* < 0.01) compared with the other groups (Figure 1(b)). To further evaluate the proliferative potential, we measured the numbers of CFUs as a function of donor age groups. On the 14th day, we counted and found an age-related decrease of CFU-forming cells in donor age groups (Figure 2).

### 3.2. Flow Cytometry Results

**Figure 3:**
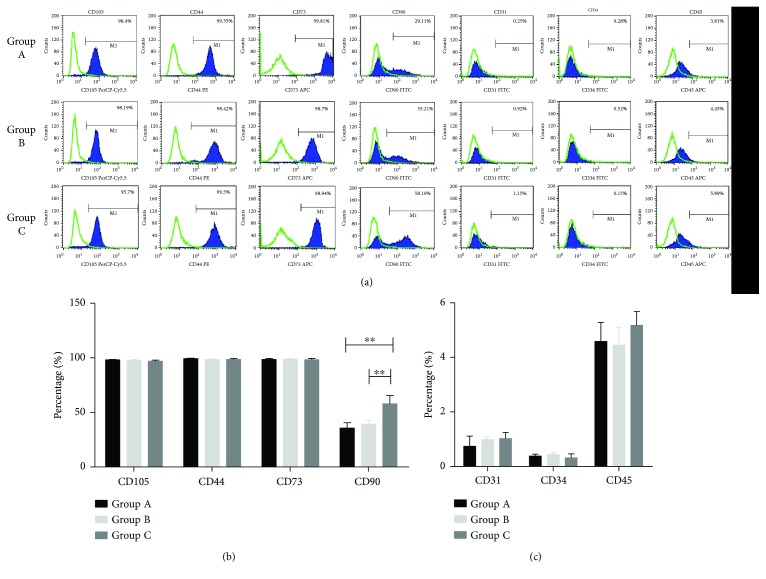
Characterization of HEASCs. (a) Flow cytometry analysis of HEASCs positive (CD105, CD44, CD73, and CD90) and negative (CD31, CD34, and CD45) surface markers. (b, c) The graph represented the percentage of specific markers in total analyzed HEASCs by all groups. Quantity of markers reveals that the expression of CD90 marker has significant differences between groups. Results showed as mean ± SD, ^∗∗^*P* < 0.01.

The expression of MSC markers was observed in HEASCs from donors of various ages by flow cytometry analysis. The HEASCs from three groups positively expressed CD105, CD44, CD73, and CD90, but little expression of CD31, CD34, and CD45 antigens (Figure 3(a)). There was a significant difference in the expression of CD90 compared to other MSCs. The expression level of CD90 (57.98 ± 7.52) in the Group C appeared significantly higher than the other groups (35.74 ± 4.87 and 39.23 ± 3.58, resp.) (Figure 3(b)). Thus, the expression of CD90 in HEASCs showed an age-related increase between the donor age groups.

### 3.3. Differentiation Potential of HEASCs

**Figure 4:**
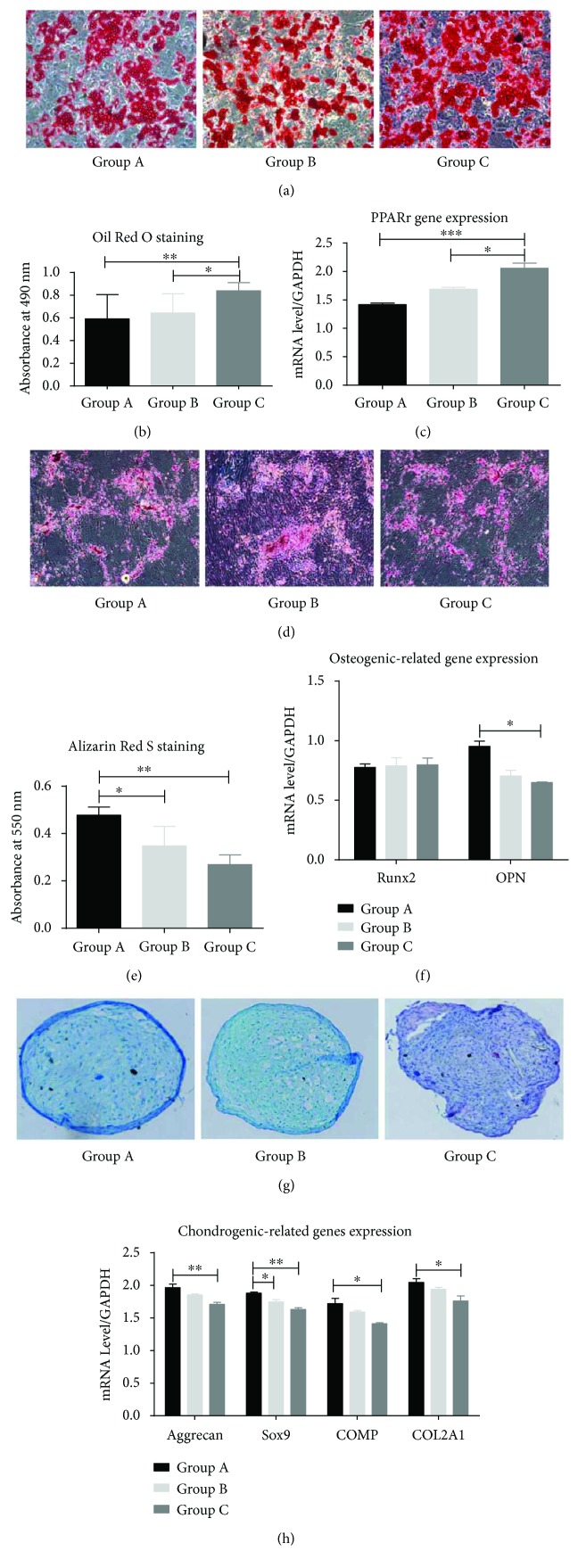
Characterization of age-related changes on the multilineage differentiation of HEASCs. (a) After 14 d in culture, the cells were stained with Oil Red O. (b) The Oil Red O solution was eluted, and the optical density was measured. (c) Gene expression analysis of PPRA*γ*. (d) After 21 d in culture, the cells were stained for calcium deposition with Alizarin Red S solution. (e) The staining solution was removed, and the absorbance was red at 550 nm. (f) The examination of the osteogenic-related gene expression of an early transcription factor (Runx2) and secreted phosphoprotein (OPN). (g) After 21 d in culture, the pellets were stained with Alcian blue and sections (6 *μ*m) were further stained with hematoxylin-eosin stain. (h) The gene expression was analyzed via Aggrecan, Sox9, COMP, and COL 2A1. Results expressed as mean ± SD. ^∗^*P* < 0.05. ^∗∗^*P* value < 0.01. ^∗∗∗^*P* value < 0.001.

In addition to proliferative ability and the expression of specific antigens on the cell surface of HEASCs, the capacity for differentiation into different mesenchymal tissues is one of the key properties of any MSC. The differentiation potential of HEASCs was evaluated by culturing them in adipogenic, osteogenic, and chondrogenic media to stimulate multilineage differentiation. After 14-day adipogenic induction in vitro, the HEASCs from all groups exhibited adipogenic differentiation potential, as determined by the presence of Oil Red-positive lipid droplets. The amount of lipid droplets was significantly increased along with the donor age (Figures 4(a) and 4(b)). Meanwhile, the gene expression of PPAR*γ* showed a higher level than that of the A and B groups (Figure 4(c)).

Osteogenic differentiation was assessed by Alizarin Red S staining and gene expression after 20 days in the osteogenic differentiation medium. Alizarin Red S staining showed a significant decrease with aging (Figures 4(d) and 4(e)), while RUNX2, an early transcription factor, has no differences. OPN, a secreted phosphoprotein, has significantly lower expression in the aged group (Figure 4(f)).

Chondrogenic differentiation was evaluated by Alcian blue staining and gene expression. Visually, the chondrogenic sections showed that the compact chondrocytes were circled by extracellular matrix in the younger donors. Conversely, the chondrocytes were loose and irregular in shape in the elderly group (Figure 4(g)). The expression of chondrogenic-related gene Aggrecan, Sox9, COMP, and COL 2A1 was consistent with Alcian blue staining (Figure 4(h)).

### 3.4. Effect of HEASCs-CM-Derived Donor Age Groups on Human Corneal Epithelial Cell Wound Healing In Vitro

**Figure 5:**
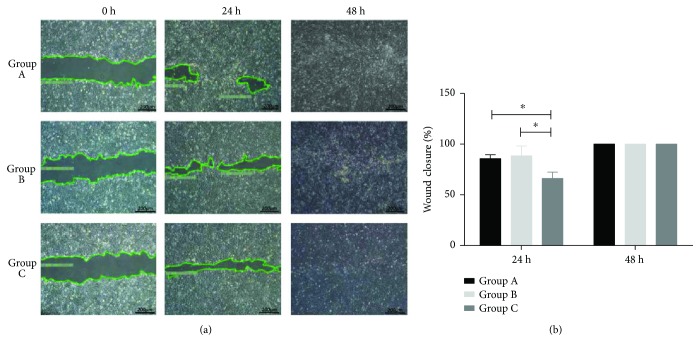
Wound healing potential of HEASCs from donors of different ages. (a) Representative figures showed human corneal epithelial cells migrated at 24 and 48 h after treatment with HEASCs-CM. (b) Changes in the wound closure area at 24 and 48 h. There was an aged-related decline in the wound healing potential of HEASCs. Data are presented as the mean ± SD. ^∗^*P* < 0.05.

To investigate whether the donor age group of HEASCs-CM impact corneal epithelial cell wound repair, we performed an in vitro wound healing assay. The HEASCs-CM significantly stimulated the rate of wound closure in Group A and Group B over Group C after 24-hour incubation; the percentage of wound closure in HEASCs-CM of these groups was 85%, 88%, and 66%, respectively. The percentage of wound closure in donor age groups was 100% after 48 h (Figures 5(a) and 5(b)). Thus, there was a significant decrease in the wound healing potential of HEASCs in advanced age group at 24 h.

### 3.5. Proteins Secreted in HEASCs-CM Encourage Wound Healing

**Figure 6:**
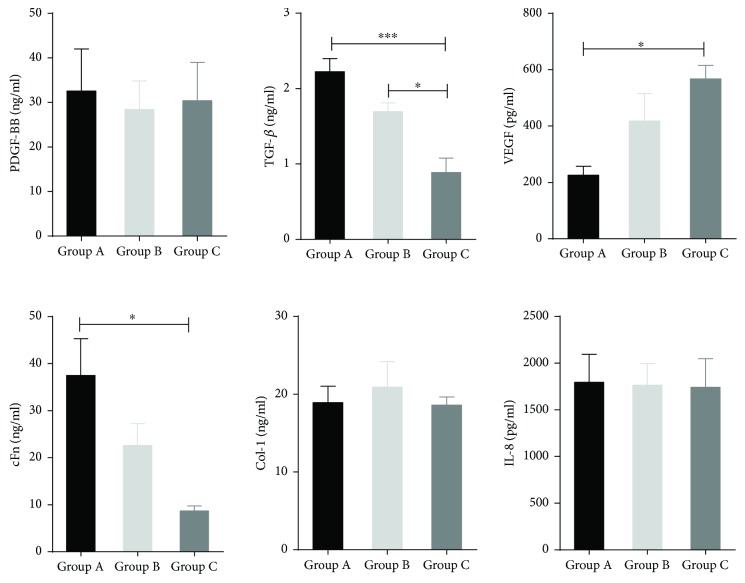
ELISA analysis of secreted proteins in HEASCs-CM. The graph showed that the expression of TGF-*β*1 and cFn was decreased with advanced age; however, the expression of VEGF demonstrated an age-related increase in donor age groups. Data was represented as mean ± SD, ^∗^*P* < 0.05. ^∗∗∗^*P* value < 0.001.

For further analysis of the wound healing potential of HEASCs of donors, we assessed the expression level of secreted wound healing-related factors in HEASCs-CM in all groups. These factors have been reported that they played an important function in wound healing, which include growth factors: PDGF-BB, TGF-*β*1, and VEGF; matrix factors: cFn and Col-1; and inflammatory cytokine: IL-8. As shown in Figure 6, the expression of TGF-*β*1 and cFn protein in HEASCs-CM was decreased with aging, while there was an exception of producing higher concentration of VEGF in the elderly group. The expression of PDGF-BB, Col-I, and IL-8 in the conditioned medium has no significant differences among these groups. These results suggested that the differences of HEASCs-CM on modulating human corneal epithelial cell wound repair might be due to the variance of expression of wound healing-related factors by HEASCs-CM.

## 4. Discussion

Adipose-derived stem cells (ASCs) are seen as useful autologous stem cells, which have gained increasing interest in the field of regenerative medicine. However, ASC therapy cannot yet be used reliably because these cells show different properties across patients and different tissue sites [19, 20]. Of note, it is an advantage to use ASCs from young donor age and of early passage culture for the clinical application of stem cell therapy; some researchers have reported the effects of donor age, long-term passage, and cryopreservation on ASC characterization, as well as proliferation and differentiation capacities in vitro [21–23]. Eyelid adipose-derived stem cells (EASCs) represent a unique origin of ASCs, which are derived from neural crest cells that differ from mesodermal-origin ASCs [7, 9]. Previous studies have disclosed that HEASCs from eyelid adipose tissue can differentiate into insulin-secreting cells and normalize type I diabetes mice [6]. In addition, the topical application of HEASCs can promote corneal tissue regeneration through paracrine effects and differentiation into corneal epithelial cells [11]. However, there is limited study demonstrating the effect of donor age on EASC properties [18].

In this study, we showed that HEASCs isolated from different donor age groups had a similar bipolar-shaped morphology by an explant culture method. However, we demonstrated that the proliferative rate of HEASCs was significantly decreased in the elderly group. Meanwhile, there was a difference in the number of CFUs in different donor age groups. The donor age-related decline of CFU number is consistent with the result that was observed in the proliferative ability of HEASCs. These results are consistent with the results that were observed in BMSCs [24].

At the surface marker expression assessment, we observed that HEASCs expressed a similar level of CD73, CD44, and CD105. However, the expression level of CD90 differed among these groups, which increased with aging. The similarity of lower expression of CD90 has been demonstrated in the previous study that most eyelid adipose-derived stem cells expressed CD90 at late passage (P13–P15) [9]. Thus, we could propose that the higher expression of CD90 in the elderly group is because of the longer time of growth in vitro in the elderly group compared to younger groups. It is noted that CD90 is membrane-bound glycoprotein, with the function related to angiogenic stimuli [25]. Meanwhile, the result was in accordance with the increase expression of VEGF in the HEASCs-CM of elderly groups (Figure 6). As we know, degenerative eye diseases, like age-related macular degeneration, are primarily a disease of old age, and VEGF (an endothelial specific growth factor) plays a key role of promoting angiogenesis and mainly influencing on eye diseases [26].

The effect of donor age on the differentiation ability of MSCs remains controversial. We found an age-related decrease in chondrogenic and osteogenic differentiations, but contrary to adipogenic differentiation potential, which was increased with aging in HEASCs. Ye et al. reported that aged orbital adipose-derived stem cells showed decreases in multilineage differentiation potential [18]. The decline of the chondrogenic differentiation potential of HEASCs is similar to that of ASC studies [27]. In contrast, we found that the HEASCs from aging promoted the adipogenic differentiation. The accumulation of adipocytes with increasing age is noted. In addition, previous studies found that ASCs from elderly donor favor the adipogenic differentiation. The author postulated that the increase of the adipogenic differentiation potential of MSCs might be negatively affected by bone regeneration [27, 28]. Although Run X2, an early transcription factor, has no differences, we observed that the expression of OPN, the gene marker of osteoblasts, declined with aging. In addition, the calcium deposits and Alizarin Red S staining were faint and lower in the aged group. Meanwhile, we clearly found an age-related decrease in chondrogenic differentiation of HEASCs (Figure 4).

Previous studies demonstrated that MSCs have gained a prominent and promising role in restoring corneal transparency through the paracrine function [29]. Thus, we evaluated the age-related effects on the wound healing potential of HEASCs by the human corneal epithelial cell scratching model in vitro. The result of this study demonstrated that the wound healing potential of HEASCs-CM declined with aging. Wound healing is a complicated process. When the cornea is damaged, the secreted protein of MSCs can accumulate at the site of injury and interact synergistically to initiate and coordinate wound healing. Therefore, we further investigated the effect of donor age on the wound healing-related protein expression in the HEASCs-CM. The results showed that the secreted protein of TGF-*β*1 and fibronectin was age related and downexpressed in the HEASCs-CM, while the expression of VEGF is upregulated with aging. TGF-*β*1 is involved in mediating the extracellular matrix formation, stimulating cell proliferation, and reepithelization. Fibronectin is involved in cell adhesion, cell motility, and wound healing. VEGF can promote angiogenesis, induce permeabilization of blood vessels, and play an essential role in wound healing. The increased expression of VEGF is consistent with the upregulation of CD90. VEGF may be involved in the degenerative disease of the elderly patient. There were no significant differences in the expression of PDGF-BB, Col-I, and IL-8, all of which were involved in the processes of wound healing [30, 31].

In conclusion, we have demonstrated that the proliferative rate, osteogenic and chondrogenic differentiation capacity, and wound healing function of HEASCs were influenced by age increase. A shift in favor of expression of CD90 surface marker and adipogenic differentiation with increased age were observed. In this study, we evaluated the effect of age on the properties of HEASCs through the protein level. To gain further insight into the molecular mechanisms, additional studies need to be performed. In particular, gene expression in HEASCs as a function of age on a genome-wide scale must be addressed to provide a comprehensive understanding of molecular mechanisms of aging.

## Figures and Tables

**Table 1:** Primers for reverse transcription-polymerase chain reaction.

Gene	Primer 5′-3′	Amplicon length (bp)
Aggrecan	F: GACTTCCGCTGGTCAGATGG	20
	R: GTTTGTAGGTGGTGGCTGTG	20

Runx-2	F: TCGGAGAGGTACCAGATGGG	20
	R: CATTCCGGAGCTCAGCAGAA	20

PPAR*γ*	F: GCAGGAGCAGAGCAAAGAG	19
	R: GAGGAGAGTTACTTGGTCGTTC	22

OPN	F: TGGGAGGGCTTGGTTGTC	18
	R: TTCCTTGGTCGGCGTTTG	18

SOX9	F: AAAGGCTACGACTGGACG	18
	R: CGGCTGGTACTTGTAATCC	19

COMP	F: CGAGTCCGCTGTATCAACACC	21
	R: TCCGTGCAAACCTGCTTGT	19

COL2	F: GCTCCCAGAACATCACCTACC	21
	R: TGAACCTGCTATTGCCCTCT	20
